# Neural Associations of Preclinical Alzheimer’s Disease in Individuals with Low Anxiety Scores

**DOI:** 10.1192/j.eurpsy.2025.287

**Published:** 2025-08-26

**Authors:** U. Cikrikcili, B. H. Schott, U. Ay, T. N. Jockschat, E. Düzel

**Affiliations:** 1 German Center for Neurodegenerative Diseases (DZNE), Magdeburg; 2 German Center for Neurodegenerative Diseases (DZNE); 3 Department of Psychiatry and Psychotherapy, University Medical Center Goettingen, Goettingen, Germany; 4 Hulusi Behçet Life Sciences Research Laboratory, Neuroimaging Unit, Istanbul University, Istanbul, Türkiye; 5 Department of Psychiatry and Psychotherapy; 6 Institute of Cognitive Neurology and Dementia Research, Otto-von-Guericke University, Magdeburg, Germany

## Abstract

**Introduction:**

Preclinical Alzheimer’s disease (AD) is characterized by subtle cognitive changes that precede the onset of clinical symptoms. Neuropsychiatric symptoms such as anxiety have been increasingly recognized for their potential role in accelerating disease progression. Although various theories have been proposed, anxiety may exacerbate cognitive decline through mechanisms involving stress-induced neurochemical dysregulation, affecting brain regions vulnerable to AD pathology.

**Objectives:**

This study examines the neural correlates of preclinical AD in individuals with low anxiety scores, employing MRI to explore potential early biomarkers and elucidate the complex role of anxiety in the progression of AD.

**Methods:**

A total of 172 participants from the German Center for Neurodegenerative Diseases Longitudinal Cognitive Impairment and Dementia Study (DELCODE) were categorized into three groups: Healthy Controls (HC, n=59), Subjective Cognitive Decline (SCD, n=77), and Mild Cognitive Impairment (MCI, n=36). Anxiety levels were assessed using the Geriatric Anxiety Inventory-Short Form (GAI-SF), and neural responses to novelty were examined using 3-Tesla MRI. Statistical models were adjusted for relevant covariates, including age, education and study site. The differences of the three groups were analysed by one-way ANOVA contrasts and post-hoc analyses were performed with two sample t-tests.

**Results:**

Significant neural differences were observed across groups, particularly in the precuneus, right angular gyrus, and right cerebellum exterior (p < 0.001, p = 0.001, and p = 0.002 respectively). The SCD group demonstrated greater activation in the right angular gyrus compared to HC (p = 0.008), while the MCI group exhibited more pronounced differences L-R precuneus, right cerebellum exterior, right angular gyrus, and right middle frontal gyrus regions indicating further cognitive decline (p< 0.001, p< 0.001, p= 0.001, p= 0.017 respectively).

**Conclusions:**

This study identifies critical brain regions, with a particular emphasis on the right angular gyrus, associated with the early stages of AD in individuals with low anxiety scores. The activation in these areas likely correlates with an early inhibition deficit at the systems level in individuals with preclinical memory impairment. However, the role of anxiety in preclinical AD is complex and variable among individuals. Anxiety may serve as an early response to subtle cognitive changes in some, while in others, it might emerge as a consequence of these changes. Moreover, the relationship between anxiety and neural alterations in AD could be bidirectional, where anxiety both influences and is influenced by the disease’s progression. These findings highlight the importance of considering anxiety when identifying early biomarkers for AD and suggest that targeted interventions addressing anxiety may help slow cognitive decline.

**Disclosure of Interest:**

None Declared

